# A prospective, longitudinal, study of men with borderline personality disorder with and without comorbid antisocial personality disorder

**DOI:** 10.1186/s40479-017-0076-2

**Published:** 2017-12-07

**Authors:** Marie-Pier Robitaille, Dave Checknita, Frank Vitaro, Richard E. Tremblay, Joel Paris, Sheilagh Hodgins

**Affiliations:** 1 Research Unit on Children’s Psychosocial Maladjustment, Université de Montréal, Montréal, Québec H1N 3M5 Canada; 2 Centre de recherche de l’Institut universitaire en santé mentale de Montréal, Montreal, Canada; 3 Departement de psychiatrie, Université de Montréal, Montreal, Canada; 4 Center for Clinical Research, Uppsala University, Västmanland County Council, Uppsala, Sweden; 5 École de psychoéducation, Université de Montréal, Montréal, Canada; 6 Département de pédiatrie, Université de Montréal, Montréal, Canada; 7 School of Public Health, Physiotherapy and Sports Science, University College Dublin, Dublin, Ireland; 8 Department of Psychiatry, McGill University, Montréal, Canada; 9 Institute of Community and Family Psychiatry, McGill University, Montréal, Canada

**Keywords:** Borderline personality disorder, Antisocial personality disorder, Crime, Psychopathic traits, Childhood behaviour, Comorbid mental health problems

## Abstract

**Background:**

Some evidence suggests that the prevalence of Borderline Personality Disorder (BPD) is elevated among male criminal offenders. It is not presently known whether offending, and violent offending, are limited to those presenting comorbid Antisocial Personality Disorder (ASPD) who have a childhood history of conduct problems and whether offending is linked to psychopathic traits.

**Methods:**

A community sample of 311 males followed from age 6 to 33 years, one third of whom had a criminal charge between ages 18 and 24, completed diagnostic interviews and the Psychopathy Checklist-Revised interview. Information on childhood included parent-reported family characteristics and teacher-rated of hurtful and uncaring behaviours, conduct problems, hyperactivity and inattention, and anxiety at age 6, 10, and 12 years. Health files were obtained as were records of criminal convictions from age 12 to 33.

**Results:**

At age 33, 4% of the men presented BPD and not ASPD, 16% ASPD and not BPD, 8% BPD + ASPD, and 72% neither disorder (ND). Comorbid disorders were common: BPD were distinguished by high levels of anxiety disorders, BPD and BPD + ASPD by depression disorders, and BPD, BPD + ASPD, and ASPD by substance dependence. Official files indicated use of health services by all participants. One-third of participants with BPD and BPD + ASPD acquired a diagnosis of a personality disorder. More than one-third of participants with BPD + ASPD obtained scores indicative of the syndrome of psychopathy. Convictions for violent crimes varied across groups: In adolescence, BPD none, BPD + ASPD 16%, ASPD 16%, and ND 3.6%; from age 18 to 33, BPD 18%, ASPD 19%, BPD + ASPD 52%, and ND 4.4%. Offenders with BPD + ASPD were convicted, on average, for four times more violent crimes than offenders with ASPD and seven times more than ND offenders. In childhood, men with BPD + ASPD and with ASPD had obtained similarly elevated ratings for disruptive behaviours as compared to ND.

**Conclusion:**

BPD comorbid with ASPD was associated with violent criminal offending in adolescence and most strongly in adulthood, elevated levels of psychopathic traits, and childhood disruptive behaviour. BPD showed similar characteristics but to a much less degree.

**Electronic supplementary material:**

The online version of this article (10.1186/s40479-017-0076-2) contains supplementary material, which is available to authorized users.

## Background

Borderline personality disorder (BPD) is characterized by interpersonal dysfunctions, affect dysregulation, impulsivity and functional disability in multiple domains. Life-time prevalence of BPD is estimated between 0.7% and 2.3% in males [1, 2]. While studies of community samples generally report no sex differences in the prevalence of BPD [3–5], fewer males than females seek treatment for BPD [6–8]. Consequently, little is known about men with BPD.

One of the major challenges in diagnosing, treating and studying BPD is the typically high levels of comorbid disorders [5], including anxiety [9, 10], depression and other mood disorders [10–13], substance use disorders [2, 4, 14, 15], suicidal and parasuicidal behaviour [13, 16] and Antisocial Personality Disorder (ASPD) [5, 17–19]. High levels of comorbidity not only make it difficult to diagnose and to treat BPD, but also to disentangle correlates and antecedents of BPD from those of comorbid disorders. Yet, such information is required to develop effective treatment and prevention programs that specifically target core mechanisms of each disorder and/or that identify mechanisms common to specific comorbidities. Further, substance misuse characterizes many males with BPD [20]. These substances lead to changes in the brain that in turn modify behaviour and further obscure identification of correlates of BPD in adulthood.

BPD is reported to be more common among male offenders than non-offenders, although diagnostic studies of prisoners have rarely assessed BPD [21]. Estimates of BPD among male offenders vary from 7.3% [22], to 19.8% [23] to 31.7% [24] in studies that used self-report questionnaires, to 26.8% from a study that used diagnostic interviews [25]. Little is known about the offences that led to incarceration of the men with BPD, whether or not they involved violence, and the age at which offending began.

Offending may be more prevalent when BPD is comorbid with ASPD (BPD + ASPD). Within correctional facilities, at least 47% of males present ASPD [21]. Studies of community samples of males presenting ASPD report that approximately one-half have been convicted of crimes, while the proportions who have engaged in physically aggressive behaviour vary from 50% to 85% [26]. However, these latter studies have not assessed comorbid BPD. In the general population, it is estimated that approximately 20% of men with BPD also present ASPD [5]. A few studies of small samples have estimated the prevalence of BPD + ASPD among offenders, with prevalence rates ranging from 10.5 to 90.9% [23, 27–29]. One study of a community sample reported more police contact and more self-reported violence among men with BPD + ASPD in comparison with men with either no disorder, BPD-only, or ASPD-only [30]*.* Studies of small clinical samples of offenders suggest that BPD + ASPD is associated with violent behaviour [30], especially if psychopathic traits are elevated [28, 31].

Given the disability associated with BPD, research has begun to focus on the identification of childhood precursors and the possibility of prevention [32]. Recent studies have identified borderline features and associated characteristics in pre-pubertal children, but have not followed participants into adulthood. For example, borderline personality traits at age 12 were associated with conduct disorder and internalizing disorders, and were preceded by poor cognitive function, impulsivity, and behaviour and emotional problems at age 5 [33]. Two studies of teenage boys identified relational aggression and depression, and not ADHD, as precursors of BPD [34, 35]. Two studies followed participants into adulthood. A community sample of adolescents followed to age 30, reported that BPD symptoms were associated with adolescent mother-child discord, depression, suicidality, maternal BPD, and paternal substance use disorder [36]. Adolescent anxiety, conduct disorder/oppositional defiant disorder, attention deficit hyperactivity disorder, and maternal substance misuse were also associated with adult BPD symptoms in univariate analyses, but were no longer significant when other risk factors were included in the model. In an assessment at age 24 of a clinical sample of males with prior disruptive behaviour disorders, BPD symptoms were associated with childhood oppositional behaviour, and not with conduct disorder, depression, or anxiety [37]. A review concluded that there is little specificity for the identified precursors for BPD, that childhood disorders such as attention deficit hyperactivity disorder, oppositional defiant disorder, conduct disorder, substance use, depression, and self-harm include symptoms analogous to BPD, but that BPD features are the most robust predictors of the disorder in adulthood. [38]. No studies have examined childhood precursors of BPD + ASPD.

### The present study

The present study aimed to further understanding of BPD, comorbid disorders, health service use, criminality, psychopathic traits, and childhood antecedents, of a community sample of males followed from age 6 to 33 years. Based on diagnoses made at age 33, four groups were compared: BPD no ASPD; BPD + ASPD, ASPD no BPD, and neither disorder (ND). Health and criminal official records were available, as were teacher ratings of behaviour at ages 6, 10, and 12.

## Methods

### Participants

Participants were drawn from the Montreal Longitudinal and Experimental Study (MLES) [39] and the Quebec Longitudinal Study of Kindergarten Children (QLSKC) [40]. These investigations recruited children of French-speaking families in the mid-1980s when they entered school. The MLES cohort includes 1037 males from a low socioeconomic status neighborhood of Montreal and the QLSKC cohort 3018 boys randomly and proportionally recruited throughout Quebec [39, 40]. The total sample included 2631 male participants. Based on official criminal records from age 12 to 24, 371 men with at least one criminal charge from age 18 to 24 and a random sample without were selected for follow-up. Multiple attempts, using multiple methods, were used to try and contact these men, and 319 completed interviews. Eight participants with severe mental illness were excluded from analyses. The final sample of 311 men were aged, on average 32.7 years (SD = 1.6), 64.8% were married, 56.9% reported having children, and 93.4% were employed. Three of the participants were included in the childhood intervention studies [41, 42].

In order to assess biases in the interviewed sample (*n* = 319), these men were compared to the 424 potential participants who were not interviewed. Results are presented in Additional file 1: Table S1. Differences in characteristics of the parents, teacher rated behaviour and academic achievement in elementary school, and adolescent delinquency, but not criminality in adulthood, indicated that the non-interviewed men were at higher risk for criminality and antisocial behaviour than the men who completed the interview.

### Procedure

The most recent addresses, telephone numbers, and email addresses were used to contact potential participants. Letters were initially sent, followed up by telephone calls and, emails inviting participation. After approval from the Commission de l’Accès à l’Information, the Regie de l’Assurance de Maladie du Québec provided addresses of potential participants. When a potential participant was contacted, the study was briefly explained, their participation requested, and if they agreed an interview was scheduled. Interviews took place at a university, participants’ homes, correctional facility, and in quiet public places. At the beginning of the interview, the study was explained to the participant, all of his questions were answered, and he signed a form consenting to any or all of the following - the interview, access to his criminal record, and access to his health record. The interviews were conducted by psychiatrists and clinical psychologists trained to use the diagnostic instruments. At the end of the interview, the participant was paid $50.00 for his time and inconvenience and travel costs.

### Ethical approval

Written consent was obtained at each wave of data collection from a parent and/or the participant (including consent regarding teachers’ reports). The study was approved by the ethics committees of the Université de Montréal, l’Hôpital Ste. Justine, and l’Institut Philippe Pinel de Montréal.

### Life-time measures

Québec has a universal, centralized, health system in which each citizen has one health file from birth to death. The files of each of the participants was obtained from the health service (Régie de l’Assurance Maladie du Québec).

### Measures in adulthood

#### Sociodemographic information

Participants reported socio-demographic information.

#### Mental Disorders

Current and lifetime axis I and II disorders were assessed using the French version of the Structured Clinical Interview for DSM-IV (SCID I and SCID II) [43, 44].

#### Psychopathy

The French version [45] of the Psychopathy Checklist Revised (PCL-R) [46] was completed based on the PCL-R interview and all the other information collected at the interview. Total and four facet scores were calculated [46]. Interrater reliability of total PCL-R scores was calculated on 21 cases. The intraclass correlation (ICC) of .872 (95% confidence interval .686–.948) indicated good inter-rater reliability.

#### Criminal convictions

Official records of criminal convictions from ages 12 to 24 were available for all participants. At age 33, 241 participants signed consents for us to obtain their criminal records from the Royal Canadian Mounted Police. Violent crimes were defined to include homicides, assaults, sexual offences, armed offences, burglary, harassment, and other crimes physically hurting people. Non-violent crimes were defined as all other crimes listed in the Canadian Criminal Code.

### Measures in childhood

Parents reported sociodemographic characteristics of the family. Official statistics were used to identify neighborhood deprivation.

#### Teacher ratings of participants’ behaviour at ages 6, 10, and 12

When participants were ages 6, 10, and 12, their classroom teachers rated behaviours (absent, sometimes, frequently) using the Social Behaviour Questionnaire [39, 40]. Cronbach alpha coefficients were calculated on the total cohort from which the sample was drawn.

#### Hurtful behaviour

Four items: tells lies; bullies others; blames others; inconsiderate of others. Cronbach alpha: age 6 .83; age 10.82; age 12 .81.

#### Uncaring behaviour

Four items were reverse coded: takes the opportunity to praise the work of less able children; shows sympathy to someone who has made a mistake; offers to help other children who are having difficulty with a task in the classroom; and comforts a youngster who is crying or upset. Cronbach Alpha: age 6 .85; age 10 .82; age 12 .81.

#### Conduct problems (CP)

Six items at age 6: destroys own or others’ belongings; fights with other children; kicks, bites, or hits other children; doesn’t share material; irritable, quick to “fly off the handle”; is disobedient. At age 10 and 12, the same items plus truant from school; has stolen things on one or more occasions. Cronbach alpha: age 6 .88; age 10 .81; age 12 .79.

#### Inattention and Hyperactivity

Six items: restless, runs about or jumps up and down, doesn’t keep still; squirmy, fidgety; has poor concentration or short attention span; inattentive; gives up easily; stares into space. Cronbach alpha: age 6 .84; age 10 .85; age 12 .86.

#### Anxiety

Five items: is worried, worries about many things; tends to do things on his own, rather solitary; appears miserable, unhappy, tearful, or distressed; tends to be fearful or afraid of new things or new situations; cries easily. Cronbach alpha: age 6 .75; age 10 .74; age 12 .75.

### Statistical analyses

Group comparisons on continuous variables were made using ANOVA and post-hoc Tukey tests, except for criminal convictions which were compared using Kruskal-Wallis and Dunn’s post-hoc tests. Comparisons on dichotomous variables were made using Fisher exact test or Pearson’s chi-square tests.

## Results

### Adulthood

Of the 311 interviewed participants, 4% (*n* = 12) presented BPD and not ASPD, 16% (*n* = 49) ASPD and not BPD, 8% (*n* = 25) BPD + ASPD, and 72% (*n* = 224) neither diagnosis (ND). As presented in Table 1, the four groups of participants did not differ as to age at the time of the interview or the proportions with children. Participants with BPD and with BPD + ASPD were less likely to be married or in a common-law marriage than participants with ASPD and ND. Participants with ASPD and BPD + ASPD were less likely to have completed high school and to be employed at the time of interview than participants with BPD and ND. BPD symptoms were similar in the BPD and BPD + ASPD groups. ASPD symptoms in the BPD + ASPD and ASPD groups were similar, although men with BPD + ASPD presented, on average, significantly more lifetime symptoms than men with ASPD.

**Table 1:** Comparisons of socio-demographic characteristics, comorbid disorders, and symptoms of men with with borderline personality bisorder, borderline personality disorder and antisocial personality disorder, antisocial personality disorder, and neither disorder on socio-demographic characteristics and comorbid disorders and symptoms

	BPD	BPD + ASPD	ASPD	ND	Statistic
N	12	25	49	224	
Sociodemographic characteristics					
Mean age at time of interview (SD)	32.20	33.51	33.16	33.15	F(3) = 1.89
	(1.53)	(1.40)	(1.61)	(1.59)	*p* = 0.131
% Married or in a common law marriage (n)	33.3	44	61.2	70	FET
	(4)	(11)	(30)	(156)	*p* = 0.005
% Completed high school (n)	75.0	28.0	44.9	84.4	X^2^(3) = 60.36
	(9)	(7)	(22	(189)	*p* < 0.001
% Employed at time of interview (n)	100.0	80.0	87.5	95.5	X^2^(3) = 10.79
	(12)	(16)	(42)	(210)	*p* = 0.013
% Report having children (n)	25.0	40.0	46.9	40.6	FET
	(3)	(10)	(23)	(91)	*p* = 0.118
Comorbid disorders					
% Anxiety disorder (n)	41.7	16.0	16.3	9.9	FET
	(5)	(4)	(8)	(22)	*p* = 0.014
% Major Depression (n)	58.3	48.1	20.4	9.5	X^2^(3) = 43.01
	(7)	(13)	(10)	(21)	*p* < 0.001
% Alcohol abuse (n)	33.3	16.0	31.3	35.7	FET
	(4)	(4)	(15)	(80)	*p* = 0.253
% Alcohol Dependence (n)	50.0	68.0	50.0	12.1	FET
	(6)	(17)	(24)	(27)	*p* < 0.001
% Drug abuse (n)	25.0	36.0	58.3	29.1	FET
	(3)	(9)	(28)	(65)	*p* = 0.002
% Drug dependence (n)	58.3	92.0	87.5	29.6	X^2^(3) = 79.64
	(7)	(23)	(42)	(66)	*p* < 0.001
Suicidal symptoms					
% Recurrent thoughts of death (n)	50.0	72.0	31.3	10.8	FET
	(6)	(18)	(15)	(24)	*p* < 0.001
% Thoughts of death (n)	33.3	64.0	30.6	11.6	FET
	(4)	(16)	(15)	(26)	*p* < 0.001
% Suicidal ideation (n)	58.3	44.0	18.4	6.3	FET
	(7)	(11)	(9)	(14)	*p* < 0.001
% Suicidal plan (n)	0.0	16.0	2.0	1.3	FET
	(0)	(4)	(1)	(3)	*p* = 0.007
% Suicide attempt (n)	16.7	20.0	8.2	1.8	FET
	(2)	(5)	(4)	(4)	*p* < 0.001

*Notes* Anovas (F), Chi-Squares (X^2^) and Fisher Exact Tests (FET) are presented. *BPD* Borderline Personality Disorder, *ASPD* Antisocial Personality Disorder, *BPD + ASPD* Borderline Personality Disorder and Antisocial Personality Disorder, *ND* neither disorder

#### Comorbid disorders

Proportionately more of the participants with BPD, than those in the other three groups, presented anxiety disorders, with and without post-traumatic stress disorder (PTSD). Greater proportions of the participants with BPD and with BPD + ASPD presented PTSD than participants with ASPD or ND. More than half the participants with BPD, and just less than half of those with BPD + ASPD, presented major depression, significantly more than participants with ASPD or ND. Additionally, large numbers of participants with BPD reported recurrent thoughts of death by suicide, suicidal ideation, and attempted suicide, and proportionately more BPD + ASPD participants, than those in other groups, reported recurrent thoughts of death (64.0%) and having had a suicide plan (16.0%).

At least one-half of the participants with BPD, BPD + ASPD, and ASPD met criteria for alcohol dependence and drug dependence. Participants with BPD + ASPD and those with ASPD presented high levels of dependence on cannabis, stimulants, cocaine and PCP, and hallucinogens.

#### Health service use

Lifetime diagnoses of hyperkinetic conduct disorder, child disturbance of emotions, conduct disorder, anxiety disorders, mood disorders, mental and behavioural disorders, substance use, adjustment disorders, overdoses and personality disorders extracted from official health files are presented in Table 2. Three-quarters of the participants with BPD and all but one of those with BPD + ASPD had acquired at least one of these diagnoses, while this was true of 57% of the ASPD and 37% of the ND. Very few of the participants had acquired diagnoses of childhood disorders. The most common diagnoses were anxiety disorders: 67% BPD and 44% BPD + ASPD and mood disorders: 66% BPD and 40% BPD + ASPD. One-third of the participants with BPD and BPD + ASPD received a diagnosis of PD, as did three of the participants with ASPD and six with ND. No participant received a diagnosis of ASPD.

**Table 2:** Comparisons of life-time diagnoses within the health system of men with borderline personality disorder, borderline personality disorder and antisocial personality disorder, antisocial personality disorder, and neither disorder

	BPD	BPD + ASPD	ASPD	ND	FET *p* value
N	12	25	49	224	
Diagnostics					
% Hyperkinetic (conduct) Disorder (n)	8.3 (1)	4.0 (1)	2.0 (1)	6.4 (14)	0.553
% Child disturbance of emotions (n)	8.3 (1)	8.0 (2)	0.0 (0)	2.3 (5)	0.093
% Conduct disorder (n)	0.0 (0)	0.0 (0)	4.1 (2)	0.9 (2)	0.291
% Anxiety (n)	66.7 (8)	44.0 (11)	32.7 (16)	26.4 (58)	0.012
% Mood disorders (n)	66.7 (8)	40.0 (10)	16.3 (8)	13.6 (30)	0.000
% Mental and behavioural disorders due to substance use (n)	33.3 (4)	60.0 (15)	24.5 (12)	7.7 (17)	0.000
% Adjustment disorders (n)	25.0 (3)	44.0 (11)	16.3 (8)	11.8 (26)	0.001
Overdoses (n)	8.3 (1)	20.0 (5)	10.2 (5)	2.7 (6)	0.002
Personality disorders (n)	33.3 (4)	32.0 (8)	8.2 (4)	2.7 (6)	0.000
Any of the above diagnosis (n)	75.0 (9)	96.0 (24)	57.1 (28)	37.3 (82)	0.000

*Notes FET* Fisher Exact Test, *BPD* Borderline Personality Disorder, *BPD + ASPD* Borderline Personality Disorder and Antisocial Personality Disorder, *ASPD* Antisocial Personality Disorder, *ND* neither disorder

#### Criminal convictions

As presented in Table 3, in adolescence, only one participant with BPD had acquired a conviction, while this was true of 40% of the men with BPD + ASPD and 39% of those with ASPD. Similar proportions (16%) of the men with BPD + ASPD and ASPD had been convicted of violent offences in adolescence. From age 18 to 33, one-third (four) of the men with BPD were convicted of a crime, as were 92% of those with BPD + ASPD and 83% of those with ASPD. While the numbers are small, similar proportions (18% and 19% respectively) of the men with BPD and of those with ASPD were convicted of violent offences, as were 52% of the men with BPD + ASPD.

**Table 3:** Comparisons of criminal convictions of participants with Borderline Personality Disorder, Borderline Personality Disorder and Antisocial Personality Disorder, Antisocial Personality Disorder, and Neither Disorder

	BPD	BPD + ASPD	ASPD	ND	Statistic	Dunn’s post-hoc (*p* value)
	a	b	c	d		
N	12	25	49	224		
Juvenile (ages 12–17)						
% at least 1 conviction (n)	8.3 (1)	40.0 (10)	38.8 (19)	12.5 (28)	FET *p* < 0.001	–
% a least 1 violent conviction (n)	0.0 (0)	16.0 (4)	16.3 (8)	3.6 (8)	FET *p* = 0.002	
% a least 1 non-violent conviction (n)	8.3 (1)	40.0 (10)	36.7 (18)	11.6 (26)	FET *p* < 0.001	
Mean (SD) number of convictions for violent crime	0.0 (0)	0.52 (1.50)	0.45 (1.44)	0.10 (0.67)	H(3) = 15.42 *p* = 0.001	c > d (0.007)
Mean (SD) number of convictions for non-violent crime	0.17 (.58)	3.04 (5.86)	1.85 (3.48)	0.52 (3.40)	H(3) = 30.41 *p* < 0.001	b > d (<0.001) c > d (0.001)
Adult (ages 18–33)						
N	11	21	42	167		
% at least 1 conviction (n)	36.4 (4)	90.5 (19)	81.0 (34)	26.3 (44)	FET *p* = 0.159	–
% at least 1 violent conviction adult (n)	18.2 (2)	52.4 (11)	19.0 (8)	4.4 (7)	FET *p* = 0.006	
% a least 1 non-violent conviction (n)	36.4 (4)	85.7 (18)	81.0 (34)	25.1 (42)	FET *p* = 0.062	
Mean (SD) number of convictions for violent crime	1.27 (3.35)	2.43 (3.64)	0.48 (1.13)	0.10 (0.49)	H(3) = 18.55 *p* < 0.001	b > a (0.028) b > c (<0.001) b > d (<0.001)
Mean (SD) number of convictions for non-violent crime	1.72 (3.58)	10.52 (15.28)	6.17 (9.37)	0.85 (2.77)	H(3) = 19.36 *p* < 0.001	b > a (0.002) b > d < 0.001 c > d (<0.001)

*Notes* Kruskal Wallis (H) are presented. Significance values have been adjusted by the Bonferroni correction. *FET* Fisher Exact Test, *BPD* Borderline Personality Disorder, *BPD + ASPD*, Borderline Personality Disorder and Antisocial Personality Disorder, *ASPD* Antisocial Personality Disorder, *ND* neither disorder

**Fig. 1:**
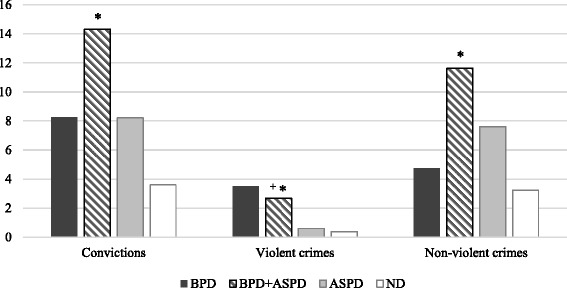
Comparisons of mean numbers of convictions of offenders with Borderline Personality Disorder, Borderline Personality Disorder and Antisocial Personality Disorder, Antisocial Personality Disorder, and neither disorder. *Notes*. *N* = 101. Dunn’s post-hoc tests with Bonferroni adjustment are presented. ^+^ = significantly different from participants with ASPD (*p* < 0.05). * = significantly different from participants with ND (*p* < 0.05)

Analyses were conducted among the 101 adult offenders. As shown in Fig. 1, the mean number of convictions for violent crimes varied across groups: BPD 3.5 (SD = 5.2), BPD + ASPD 2.68 (SD = 3.7), ASPD .59 (SD = 1.2), ND .36 (SD = .9) (H(3) = 14.90, *p* = .002). BPD + ASPD offenders had been convicted, on average, for four times more violent crimes than offenders with ASPD (*p* = 0.026) and seven times more than ND offenders (*p* = 0.002). BPD offenders had been convicted, on average, for approximately six times more violent crimes than offenders with ASPD and almost ten times more than ND offenders, although these differences were not statistically significant. Only one significant difference emerged in comparisons of numbers of convictions for non-violent crimes: offenders with BPD + ASPD were convicted three times more frequently for non-violent crimes than ND participants (*p* = 0.016).

#### Psychopathic traits

As presented in Table 4, participants with BPD obtained higher scores than those with ND only on facet 3 (lifestyle) of the PCL-R. By contrast, those with BPD + ASPD obtained higher total and facet scores than ND, as well as higher total and facets 2 (affective), 3 (lifestyle), and 4 (antisocial) scores than ASPD participants. Because of small and unequal group sizes, effect sizes must be interpreted with caution. Partial Eta-squared were, however, estimated and suggest moderate to large effect size. Cohen’s ds were also calculated in two-by-two group comparisons and similarly suggested large effect sizes (see Additional file 1). More than one-third of participants with BPD + ASPD obtained scores of 30 or higher indicative of the syndrome of psychopathy, while this was true for none of the BPD participants and 12% of those with ASPD.

**Table 4:** Comparisons of psychopathy checklist-revised scores of men with borderline personality disorder, borderline personality disorder and antisocial personality disorder, antisocial personality disorders, and neither disorder

	BPD	BPD+ ASPD	ASPD	ND	Statistic	Tukey’s HSD p value	Partial Eta Squared
	a	b	c	d			
N	12	25	49	224			
Mean Total PCL-R Score (SD)	7.83 (7.60)	23.08 (10.30)	15.22 (8.90)	3.11 (4.92)	F(3) = 108.24 p = <0.001	a < b 0.002 a < c < 0.001 b > c < 0.001 b > d < 0.001 c > d < 0.001	0.52
Mean Facet 1: Interpersonal (SD)	1.25 (1.48)	3.24 (2.60)	2.53 (2.26)	0.70 (1.35)	F(3) = 29.85 p = <0.001	a < b 0.004 b > d < 0.001 c > d < 0.001	0.23
Mean Facet 2: Affective (SD)	2.0 (1.91)	5.88 (2.15)	4.35 (2.29)	0.77 (1.53)	F(3) = 107.12 p = <0.001	a < b 0.002 a < c < 0.001 b > c 0.002 b > d < 0.001 c > d < 0.001	0.51
Mean Facet 3: Lifestyle (SD)	2.58 (2.50)	6.12 (2.96)	3.82 (2.67)	0.78 (1.42)	F(3) = 85.88 p = <0.001	a < b < 0.001 a > d 0.007 b > c < 0.001 b > d < 0.001 c > d < 0.001	0.46
Mean Facet 4: Antisocial (SD)	1.33 (2.81)	5.88 (3.30)	3.51 (3.01)	0.57 (1.59)	F(3) = 65.55 p = <0.001	a < b < 0.001 a < c 0.008 b > c < 0.001 b > d < 0.001 c > d < 0.001	0.39
% Scores 30+ on PCL-R (n)	0.0 (0)	36.0 (9)	12.2 (6)	0.9 (2)	FET P < 0.001	–	

*Notes* Anovas are presented (F). *FET* Fisher Exact Test, *BPD* Borderline Personality Disorder, *BPD + ASPD* Borderline Personality Disorder and Antisocial Personality Disorder, *ASPD* Antisocial Personality Disorder, *ND* neither disorder

### Childhood

#### Family characteristics

Participants from the four groups did not differ as to maternal employment, deprived neighborhood nor family income, while participants with BPD + ASPD had younger mothers than participants with ND (mean age 22.76, *SD* = 3.73 vs. 25.81, *SD* = 4.66; Additional file 1: Table S2). Compared with the other groups, proportionally more participants with BPD + ASPD had a mother or a father with a criminal conviction (16% and 20%, respectively), whereas proportionately more participants with ASPD had fathers with criminal convictions.

#### Teacher ratings of participants’ behaviour

**Fig. 2:**
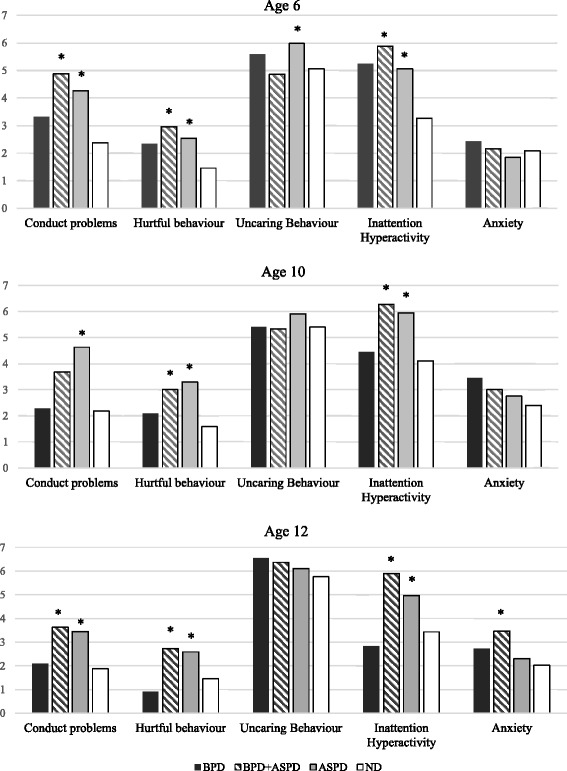
Comparisons of teacher ratings at ages 6, 10, and 12 of men with Borderline Personality Disorder, Antisocial Personality Disorder, Borderline Personality Disorder and Antisocial Personality Disorder, and neither disorder. *Notes*. * = significantly different from participants with ND (*p* < 0.05)

Results are presented in Fig. 2 and Additional file 1: Table S2. Post-hoc tests indicated that participants with BPD did not differ from those with ND on any ratings at any age. By contrast, those with BPD + ASPD obtained higher ratings than ND for CP at ages 6 and 12, hurtful behaviours and inattention and hyperactivity at ages 6, 10 and 12, and anxiety at age 12. Participants with BPD + ASPD obtained similar ratings as those with ASPD for almost all childhood behaviours at each age, with two exceptions: as compared to ND, BPD + ASPD obtained ratings for CP at age 10 that were higher but did not differ statistically whereas participants with ASPD obtained significantly higher ratings; and participants with BPD + ASPD obtained higher ratings than ND for anxiety at age 12 while participants with ASPD did not.

## Discussion

The present study prospectively followed 311 men from age 6 to 33 and investigated BPD with and without comorbid ASPD. Overall, the prevalence of BPD was 11.9%, much higher than previous reports from community samples [47][3, 5]. Importantly, 68% of the men with BPD also presented ASPD consistent with a few previous reports [18, 19]. The prevalence of ASPD was twice as high as that of BPD + ASPD.

There was no difference in the prevalence of BPD-only among convicted offenders (3.2%) and non-offenders (4.4%), but 17.7% of the offenders and only 1.7% of the non-offenders presented BPD + ASPD. This finding suggests that previous studies reporting high rates of BPD among offenders [22–24, 48]may not have diagnosed comorbid ASPD. As would be expected, one-third of the offenders and only 5.0% of the non-offenders presented ASPD. Four key findings emerged.

One, BPD was associated with violent crime in adulthood among men with no history of violent crime in adolescence. Similar proportions of men with BPD + ASPD and ASPD had acquired juvenile criminal records for non-violent and violent crime. However, from age 18 to 33, 52% of those with BPD + ASPD and only 19% of those with ASPD acquired convictions for violent crimes. A similar increase in violence with age was observed among the men with BPD. Although two men with BPD had acquired convictions for violence in adulthood, neither had been convicted for violence in adolescence. These findings suggest that among the men with BPD and those with BPD + ASPD a change in self-regulation occurred in the transition to adulthood leading to levels of violent convictions greater than those observed among the men with ASPD. Previous studies proposed that violence among men with BPD + ASPD may be mediated by alcohol misuse [27]. In the present study, however, life-time diagnoses of alcohol dependence were acquired by with BPD + ASPD, as well as those with ASPD and those with BPD. At ages 6, 10, and 12 both men with BPD + ASPD and those with ASPD, but not those with BPD, were rated by different teachers as showing higher levels of conduct problems, hurtful behaviour, and inattention/hyperactivity than those with ND, and in adolescence 16% of both ASPD groups and none of the BPD were convicted for violent offences. Yet, in adulthood offenders with BPD + ASPD were convicted for violence almost five times more frequently than offenders with ASPD, and those with BPD six times more frequently. These results suggest that among men with BPD, whether or not it is comorbid with ASPD, for some unknown reason, the transition to adulthood is associated with an increased risk of violence.

The pattern of violent offending observed among the men with ASPD + BPD and BPD is strikingly different from the pattern observed among the ASPD that shows continuity from childhood with elevated ratings of conduct problems, hurtful behaviours, and inattention/hyperactivity, juvenile offending, and adult offending. This pattern of stable antisocial behaviour from childhood onwards has been robustly documented in prospective studies [49–53]. Among the men with ASPD-only, 37% were convicted for non-violent crimes in adolescence, and 69% in adulthood. This finding concurs with previous studies of ASPD showing elevated rates of non-violent offending, and lower rates of violent offending [26]. Consequently, the high rate of violent offending in adulthood of men with BPD + ASPD and those with BPD is different from that typically observed among men with ASPD.

A second key finding was the significantly higher levels of psychopathic traits among men with BPD + ASPD than among those with ASPD. The men with BPD + ASPD obtained higher total PCL-R scores, and facets 2, 3, and 4 scores than the men with ASPD. Further, one-third of the men with BPD + ASPD as compared to 12% of those with ASPD met criteria for the syndrome of psychopathy. This is a curious finding since BPD is characterized by emotional lability and psychopathy by low levels of emotion, and indeed, the men with BPD + ASPD obtained higher scores on the affective facet of psychopathy than either men with ASPD or those with BPD. However, one study of adult male violent offenders with high PCL-R scores identified two sub-groups, one presenting high levels of trait anxiety and borderline personality features [54]. A similar sub-group was also identified among adolescents with high psychopathic trait scores [55]. Importantly, among three-year old children, a sub-group presenting high levels of callousness, externalizing, and internalizing behaviours was identified and these characteristics remained stable into adolescence [56]. Psychopathic traits have been shown to emerge in early childhood [57] and to remain relatively stable from childhood through early adulthood [58], and thus it is difficult to understand why and how they would contribute to an increase in violent offending in adulthood and not earlier.

In another study of adult offenders, impulsive aggression was associated with the sum of facet 3 and 4 scores only among those with generalized anxiety disorder [59]. Such offenders may present BPD + ASPD. However, in the present study, anxiety disorders were more common among the men with BPD (42%), than among men with BPD + ASPD (16%) and those with ASPD (16%) suggesting that fear was higher in BPD and was attenuated among those with comorbid ASPD. By contrast, major depression, thought to index distress, was diagnosed among 58% of BPD, 48% of BPD + ASPD, and 20% of ASPD, suggesting an association with BPD regardless of ASPD, consistent with previous findings indicating that distress is a key feature of BPD [60].

A third key finding from the present study was that the men with BPD + ASPD showed a similar profile of childhood behaviour problems as did men with ASPD, and significantly different than men with ND. Importantly, however, our study included no measures specific to BPD. Classroom teachers rated participants with BPD + ASPD and ASPD similarly at ages 6, 10, and 12, on conduct problems, inattention and hyperactivity, known predictors of antisocial behaviour in adulthood, and on hurtful and uncaring behaviours, thought to be antecedents of psychopathic traits. Generally, the ratings for the BPD fell between those for the BPD + ASPD and ASPD and the ND. These findings are consistent with results of studies of children and adolescents showing that those presenting BPD features presented elevated rates of conduct disorder [33]. Yet few of the participants in the present study were recognized by the health system as presenting either externalizing or internalizing problems in childhood. Conduct problems in children are reduced when their parents complete parenting programs [61, 62], the antecedents of psychopathy are reduced by warm, optimal parenting [63], and when parents complete specific parenting programs [64]. Future research is needed to determine whether such interventions could prevent the development of BPD + ASPD. However, in the present study, more than one-third of the BPD + ASPD had a parent with a criminal record. Antisocial parents are known to provide non-optimal parenting and to have children with conduct problems [65], and they may be resistant to participating in parent-training programs.

A fourth key finding was that only one-third of the men with BPD and BPD + ASPD were identified by the health system as having a personality disorder and thus could not access BPD treatment programs. Further, only 8% of the men with ASPD received a diagnosis of a personality disorder. A recent report of Quebec health system data concluded that a diagnosis of a personality disorder is given only when it is considered the primary disorder [66]. Results suggest that when the men with BPD and ASPD + BPD did contact the health system, comorbid disorders were viewed as primary. Despite teacher ratings indicative of childhood disorders, no participant with BPD or with BPD + ASPD had acquired a diagnosis of conduct disorder, and only two a diagnosis of Attention Deficit Hyperactivity Disorder.

### Strengths and limitations

The principal strength of the present study was the data prospectively collected over 27 years of a relatively large sample of males. Another strength was the use of structured and validated instruments administered by clinicians trained specifically to use these instruments to diagnose mental disorders and assess psychopathic traits. Different classroom teachers at age 6, 10, and 12 provided ratings of behaviours. A final strength was the availability of official juvenile and adult criminal records and health records.

The principal weakness of the study was the large proportion of cohort members who did not complete the age 33 follow-up. Comparisons of those who did and did not complete the follow-up showed that the interviewed participants were characterized by lower levels of disruptive behaviours in childhood, and less delinquency in adolescence than the non-interviewed. Consequently, findings likely underestimate the association of BPD with antisocial behaviour. Despite this bias in the interviewed sample, meaningful associations with antisocial behaviour and crime were identified. Another limitation was the absence of measures of BPD features in childhood. This prospective, longitudinal, study was designed and established in the early 1980’s when there was little knowledge or theorizing about the childhood origins of BPD. Consequently, most of the childhood ratings focused on behaviour problems that were thought to lead to antisocial behaviour and/or criminality. Another weakness of the study was the absence of information about maltreatment in childhood. The small number of participants with BPD did not allow multivariate analyses.

## Conclusions

The present study examined 311 males followed from age 6 to 33. By age 24, one-third had acquired at least one criminal charge, and by age 33, of the 241 who consented to a criminal record check, 40.5% had acquired a criminal record. Diagnostic interviews revealed that 11.9% of the men met criteria for BPD, and two-thirds of them, also presented ASPD. BPD, with and without comorbid ASPD, was associated with convictions for violent crime more strongly than ASPD, especially in adulthood. Further, BPD comorbid with ASPD was associated with elevated levels of psychopathic traits, anxiety, major depression, alcohol, and drug dependence. Health system files indicated that only one-third of the men with BPD or BPD + ASPD had been diagnosed with a personality disorder thereby making them eligible for treatment programs for BPD. In elementary school, the boys developing BPD + ASPD and ASPD presented behavioural antecedents of antisocial behaviour and psychopathic traits. Yet, few had been recognized by the health system as presenting either internalizing or externalizing disorders in childhood. Given recent evidence demonstrating the effectiveness of optimal parenting in reducing these antecedents, research is urgently needed to trial childhood interventions aimed at preventing the development of BPD.

## Additional file

Additional file 1: Table S1. Comparison of characteristics of interviewed and non-interviewed men. **Table S2.** Comparisons of parent characteristics and teacher ratings at ages 6, 10, and 12 of men with Borderline Personality Disorder, Borderline Personality Disorder and Antisocial Personality Disorder, Antisocial Personality Disorder, and Neither Disorder. **Table S3.** Estimation of effect size of group differences in PCL-R scores. **Table S4.** PCL-R Scores of Each Group of the Study. (DOCX 46 kb)

## Abbreviations

ASPD: Antisocial personality disorder
BPD: Borderline personality disorder
DSM-IV: Diagnostic and statistical manual of mental disorders – Fourth edition
MLES: Montreal longitudinal and experimental study
ND: Neither disorder
PCL-R: Psychopathy checklist - revised
QLSKC: Quebec longitudinal study of kindergarten children
SCID: Structured clinical interview for DSM
