# Supplementary material for: A prospective, longitudinal, study of men with borderline personality disorder with and without comorbid antisocial personality disorder
Source: Borderline Personal Disord Emot Dysregul. 2017 Dec 7;4:25. doi: 10.1186/s40479-017-0076-2 (PMC5719590; doi:10.1186/s40479-017-0076-2)
Supplement: Additional file 1: Table S1. — Comparison of characteristics of interviewed and non-interviewed men. Table S2. Comparisons of parent characteristics and teacher ratings at ages 6, 10, and 12 of men with Borderline Personality Disorder, Borderline Personality Disorder and Antisocial Personality Disorder, Antisocial Personality Disorder, and Neither Disorder. Table S3. Estimation of effect size of group differences in PCL-R scores. Table S4. PCL-R Scores of Each Group of the Study. (DOCX 46 kb) [file 40479_2017_76_MOESM1_ESM.docx]

**Supplementary on-line material**

A Prospective, Longitudinal, Study of Men with Borderline Personality Disorder With and Without Comorbid Antisocial Personality Disorder

Marie-Pier Robitaille, Dave Checknita, Frank Vitaro, Richard E. Tremblay, Joel Paris, & Sheilagh Hodgins

Table S1. Comparison of characteristics of interviewed and non-interviewed men

|  |  | **Interviewed** | **Not-interviewed** | **Statistic** |
| --- | --- | --- | --- | --- |
| N |  | 319 | 424 |  |
| **Environmental characteristics** | | | | |
| % (n) Neighborhood wealth (poor) |  | 41.7  (133) | 42.2  (179) | X²(1) = 0.02  *p* = 0.886 |
| **Parents characteristics** | | | | |
| % (n) Single parent |  | 25.1  (78) | 33.0  (140) | X²(1) = 7.34  ***p* = 0.007** |
| Mother’s mean age at participant’s birth (SD) |  | 25.32  (4.66) | 25.39  (4.78) | *t*(728) = 0.20  *p* = 0.839 |
| % (n) Mother employed at child age 6 |  | 45.5  (143) | 37.8  (151) | X²(1) = 4.30  ***p* = 0.038** |
| % (n) Mother with little education |  | 33.4  (104) | 42.7  (181) | X²(1) = 9.98  ***p* = 0.002** |
| % (n) Mother with criminal record |  | 4.1  (13) | 7.1  (30) | X²(1) = 3.005  *p* = 0.083 |
| % (n) Father with criminal record |  | 9.1  (29) | 17.2  (73) | X²(1) = 10.15  ***p* = 0.001** |
| **Teachers’ ratings mean scores (SD) – age 6** | | | | |
| Hurtful behaviour |  | 1.82  (2.05) | 2.31  (2.21) | *t*(699) = 3.09  ***p* = 0.002** |
| Uncaring behaviour |  | 5.24  (1.99) | 5.37  (2.02) | *t*(736) = 0.82  *p* = 0.415 |
| Conduct problems |  | 2.95  (3.21) | 3.65  (3.34) | *t*(741) = 2.87  ***p* = 0.004** |
| Anxiety |  | 2.06  (2.23) | 2.05  (2.17) | *t*(741) = -0.10  *p* = 0.917 |
| Hyperactivity and Inattention |  | 3.86  (3.37) | 4.72  (3.19) | *t*(741) = 3.55  ***p* < 0.001** |
| **Teachers’ ratings mean scores (SD) – age 10** | | | | |
| Hurtful behaviour |  | 2.03  (2.29) | 2.49  (2.33) | *t*(633) = 2.49  ***p* = 0.013** |
| Uncaring behaviour |  | 5.46  (2.05) | 5.60  (1.94) | *t*(629) = 0.87  *p* = 0.385 |
| Conduct problems |  | 2.72  (3.09) | 3.24  (3.30) | *t*(639) = 2.01  ***p* = 0.045** |
| Anxiety |  | 2.53  (2.25) | 2.57  (2.37) | *t*(639) = 0.24  *p* = 0.814 |
| Hyperactivity and Inattention |  | 4.62  (3.59) | 5.42  (3.46) | *t*(639) = 2.85  ***p* = 0.004** |
| **Teachers’ rating mean scores (SD) – age 12** | | | | |
| Hurtful behaviour |  | 1.73  (2.12) | 2.17  (2.13) | *t*(585) = 2.50  ***p* = 0.013** |
| Uncaring behaviour |  | 5.90  (1.72) | 6.07  (1.79) | *t*(589) = 1.14  *p* = 0.254 |
| Conduct problems |  | 2.31  (2.77) | 2.73  (2.77) | *t*(593) = 1.87  *p* = 0.063 |
| Anxiety |  | 2.19  (2.15) | 2.39  (2.27) | *t*(593) = 1.10  *p* = 0.272 |
| Hyperactivity and Inattention |  | 3.88  (3.58) | 5.01  (3.37) | *t*(593) = 3.97  ***p* < 0.001** |
| Writing marks |  | 2.93  (1.23) | 2.49  (1.18) | *t*(585) = -4.39  ***p* < 0.001** |
| Reading marks |  | 3.09  (1.16) | 2.67  (1.16) | *t*(586) = -4.37  ***p* < 0.001** |
| Mathematics marks |  | 3.29  (1.24) | 2.88  (1.19) | *t*(586) = -4.09  ***p* < 0.001** |
| **Juvenile criminal records (age 12 to 17)** | | | | |
| % (n) with criminal convictions |  | 19.4  (62) | 31.6  (134) | X²(1) = 13.88  ***p* < 0.001** |
| Mean (SD) number of criminal convictions |  | 0.10  (0.67) | 0.12  (0.63) | *t*(675.5) = 4.02  ***p* < 0.001** |
| **Adult criminal records (age 18 to 24)** | | | | |
| % (n) with criminal convictions |  | 33.5  (107) | 39.4  (167) | X²(1) = 2.67  *p* = 0.102 |
| Mean (SD) number of criminal convictions |  | 1.17  (3.21) | 1.44  (3.13) | *t*(741) = 1.20  *p* = 2.229 |

Notes. Valid percentages. Pearson’s chi-square tests and T-tests are presented. Data were available for more than 94% of both samples, except for age 10 and 12 teachers’ rating, when they were available for over 85% and 77%, respectively.

Table S2. Comparisons of parent characteristics and teacher ratings at ages 6, 10, and 12 of men with Borderline Personality Disorder, Borderline Personality Disorder and Antisocial Personality Disorder, Antisocial Personality Disorder, and Neither Disorder

|  | BPD | ASPD | BPD+ASPD | ND | **Statistic** | **Tukey’s HSD**  ***p* value** |
| --- | --- | --- | --- | --- | --- | --- |
|  | **a** | **b** | **c** | **d** |  |  |
| N | 12 | 49 | 25 | 224 |  |  |
| **Family characteristics** | | | | | |  |
| Mean age of mother at birth (SD) | 23.83  (4.10) | 24.39  (4.54) | 22.76  (3.73) | 25.81  (4.66) | F(3)=4.60  *p* = 0.004 | c<d 0.009 |
| % Maternal employment outside home, age 6 (n) | 58.3  (7) | 43.8  (21) | 40  (10) | 46.2  (102) | X²(3)=1.18  *p* = 0.756 |  |
| % Deprived neighborhood (n) | 25.0  (3) | 42.9  (21) | 48  (12) | 41.5  (93) | FET  *p* = 0.63 |  |
| % Family income above $15,000, age 10 (n) | 100  (9) | 79.4  (27) | 68.8  (11) | 86.3  (145) | X²(3)=5.77  *p* = 0.123 |  |
| % Mother with criminal convictions | 0 | 2.0  (1) | 16  (4) | 2.2  (5) | FET  *p* = 0.02 |  |
| % Father with criminal convictions | 8.3  (1) | 14.3  (7) | 20  (5) | 6.3  (14) | FET  *p* = 0.04 |  |
| **Teacher’s ratings – age 6** | | | | | |  |
| Conduct problems mean score (SD) | 3.33  (3.39) | 4.27  (3.38) | 4.88  (4.06) | 3.38  (2.85) | F(3)=8.93  *p* < 0.001 | b>d 0.001  c>d 0.001 |
| Hurtful behaviour mean score (SD) | 2.33  (2.77) | 2.54  (2.27) | 2.96  (2.48) | 1.46  (1.73) | F(3)=7.91  *p* < 0.001 | b>d 0.003  c>d 0.002 |
| Uncaring behaviour mean score (SD) | 5.58  (2.19) | 5.98  (1.81) | 4.87  (1.71) | 5.06  (2.03) | F(3)=3.28  *p* = 0.021 | b>d 0.018 |
| Inattention hyperactivity mean score (SD) | 5.25  (3.93) | 5.06  (3.02) | 5.88  (3.75) | 3.26  (3.17) | F(3)=8.83  *p* < 0.001 | b>d 0.003  c>d 0.001 |
| Anxiety mean score (SD) | 2.42  (2.97) | 1.84  (2.21) | 2.16  (2.61) | 2.08  (2.14) | F(3)=0.29  *p* = 0.834 |  |
| **Teacher’s rating – age 10** | | | | | |  |
| Conduct problems mean score (SD) | 2.27  (2.33) | 4.64  (3.39) | 3.68  (2.71) | 2.18  (2.89) | F(3)=9.28  *p* < 0.001 | b>d <0.001 |
| Hurtful behaviour mean score (SD) | 2.09  (2.39) | 3.30  (2.31) | 3.00  (2.33) | 1.58  (2.10) | F(3)=9.28  *p* < 0.001 | b>d <0.001  c>d 0.020 |
| Uncaring behaviour mean score (SD) | 5.40  (2.17) | 5.91  (1.90) | 5.33  (2.54) | 5.40  (2.01) | F(3)=.79  *p* = 0.50 | - |
| Inattention hyperactivity mean score (SD) | 4.45  (3.30) | 5.95  (3.69) | 6.27  (3.59) | 4.11  (3.49) | F(3)=5.05  *p* = 0.002 | b>d 0.011  c>d 0.035 |
| Anxiety mean score (SD) | 3.45  (2.25) | 2.75  (2.24) | 3.00  (2.39) | 2.40  (2.24) | F(3)=1.268  p=0.286 | - |
| **Teacher’s rating – age 12** | | | | | |  |
| Conduct problems mean score (SD) | 2.09  (1.92) | 3.45  (3.17) | 3.63  (2.50) | 1.87  (2.59) | F(3)=5.93  *p* = 0.002 | b>d 0.003  c>d 0.033 |
| Hurtful behaviour mean score (SD) | .91  1.04 | 2.59  2.12 | 2.72  2.72 | 1.45  2.01 | F(3)=5.67  *p* = 0.001 | b>d 0.006  c>d 0.062 |
| Uncaring behaviour mean score (SD) | 6.55  1.86 | 6.11  1.60 | 6.37  1.57 | 5.76  1.75 | F(3)=.79  *p* = 0.500 | - |
| Inattention hyperactivity mean score (SD) | 2.82  2.56 | 4.97  3.41 | 5.89  3.46 | 3.43  3.55 | F(3)=4.89  *p* = 0.003 | b>d 0.045  c>d 0.019 |
| Anxiety mean score (SD) | 2.73  (1.95) | 2.30  (1.75) | 3.47  (2.22) | 2.02  (2.21) | F(3)=2.973  *p* = 0.032 | c>d 0.025 |

*Notes*. Anovas are presented (F). FET = Fisher Exact Test. BPD = Borderline Personality Disorder. ASPD = Antisocial Personality Disorder, BPD+ASPD = Borderline Personality Disorder and Antisocial Personality Disorder. ND = neither disorder.

Table S3. Estimation of effect size of group differences in PCL-R scores.

|  |  | *M* | *SD* | *df* | *t* | *p* | Cohen’s *d* |
| --- | --- | --- | --- | --- | --- | --- | --- |
| VS ND | | 3.11 | 4.92 |  |  |  |  |
|  | **BPD only** | 7.83 | 7.60 | 233 | 3.14 | 0.002 | 0.74 |
|  | **ASPD only** | 15.22 | 8.90 | 270 | 13.17 | < 0.001 | 1.68 |
|  | **BPD+ASPD** | 23.08 | 10.30 | 25.242 | 9.574^a^ | < 0.001 | 2.48 |
| VS BPD+ASPD | | 23.08 | 10.30 |  |  |  |  |
|  | **BPD** | 7.83 | 7.60 | 35 | -4.55 | < 0.001 | 1.68 |
|  | **ASPD** | 15.22 | 8.90 | 72 | -3.40 | 0.001 | 0.82 |

*Notes*. ^a^equal variance not assumed (significant Levene Test).

Table S4. PCL-R Scores of Each Group of the Study.

|  | No BPD | BPD |
| --- | --- | --- |
| No ASPD | 3.11  (4.92) | 7.83  (7.60) |
| ASPD | 15.22  (8.90) | 23.08  (10.30) |

*Notes.* PCL-R mean scores and standard deviations () are presented.
